# Supplementary material for: Longitudinal viral shedding and antibody response characteristics of men with acute infection of monkeypox virus: a prospective cohort study
Source: Nat Commun. 2024 May 27;15:4488. doi: 10.1038/s41467-024-48754-8 (PMC11130326; doi:10.1038/s41467-024-48754-8)
Supplement: Supplementary file 1 — Supplementary Information [file 41467_2024_48754_MOESM1_ESM.pdf]

Figure S1. Sensitivity of the MPXV specific qRT- PCR assay in our study using standard plasmid. A: Representative amplification plots of the 10-fold diluted standard plasmid. The numbers near the amplification plots represent the copies of standard plasmid (copies/μl). B: Standard curve for 10-fold serial dilutions of the standard plasmid.

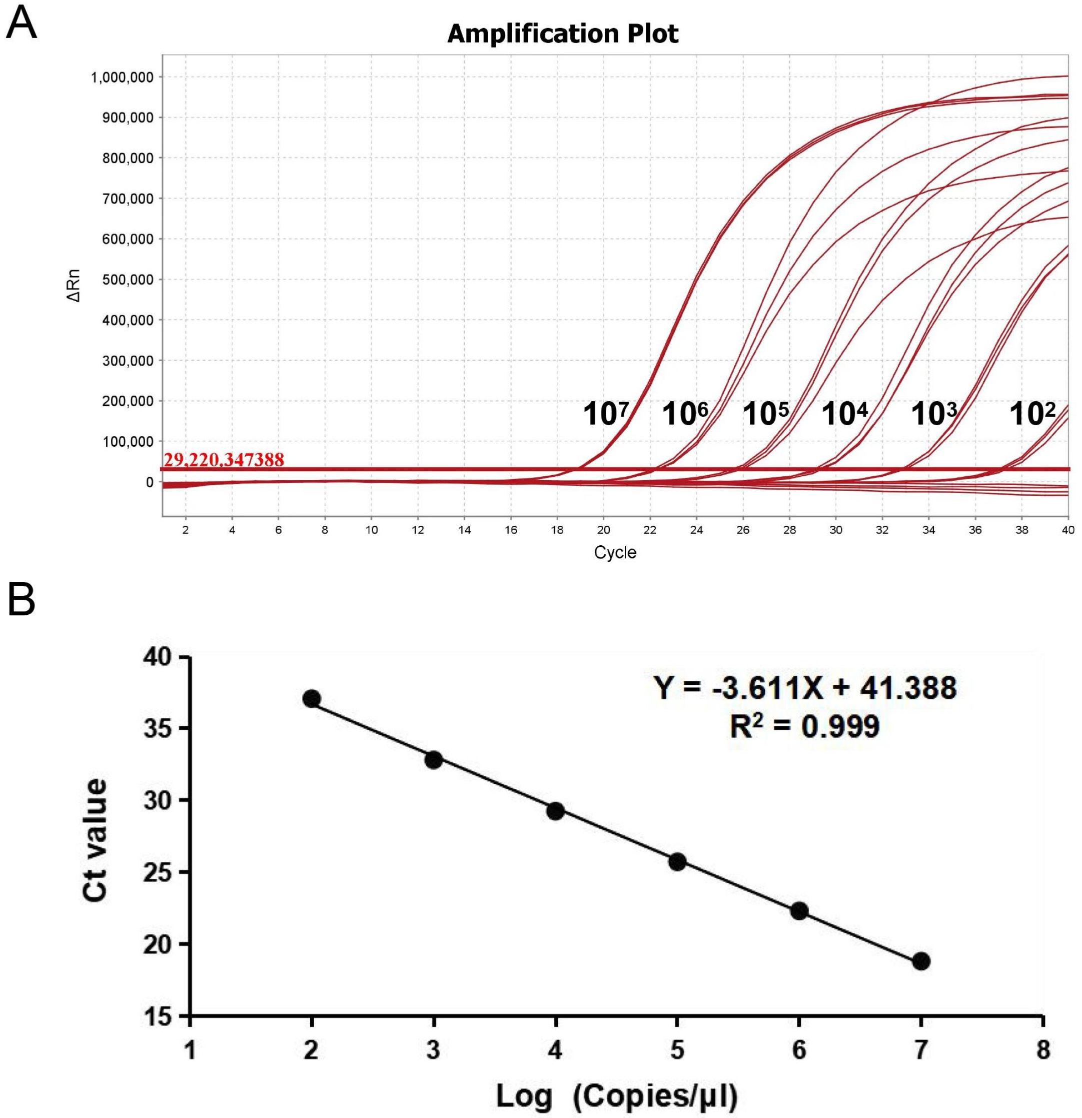

Figure S2. Temporal distributions of Mpox patients in Shenzhen during June 11, 2023 and November 13, 2023.

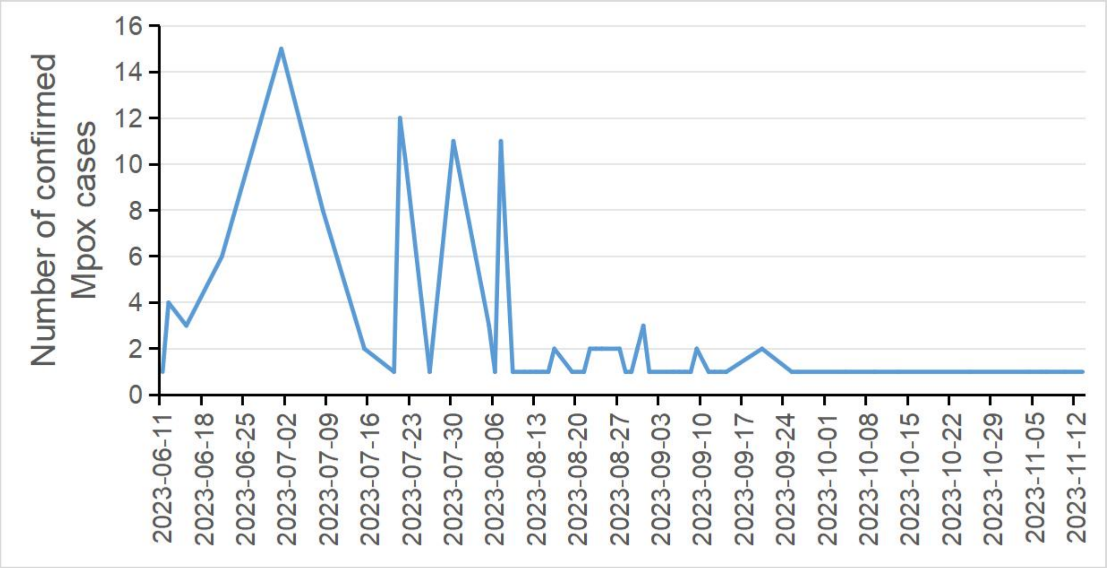

Figure S3. Comparison of the peak viral load in skin lesions (A), rectal swab (B), saliva (C) and oropharyngeal swabs (D) between HIV (N=42) and Non-HIV (N=35) participants. Viral load is expressed as $\log_{10}$ copies per mL. Statistical significance was measured using a Mann-Whitney two-sided test U-test, and p values less than 0.05 were considered statistically significant.

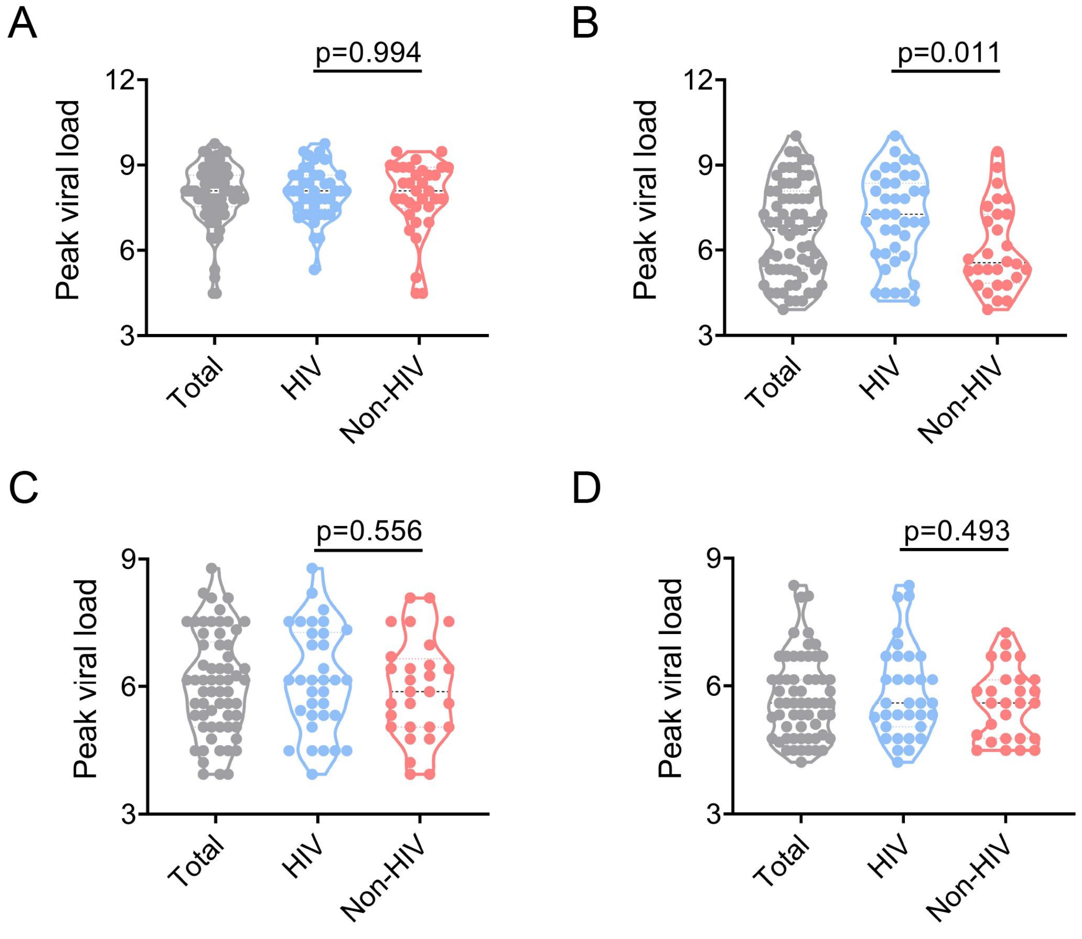

Figure S4. The influence of HIV and rectitis and the peak viral loads and viral shedding dynamics of MPXV in rectal swabs. A and B: Comparison of the peak viral load (A) and viral shedding dynamics (B) of MPXV in rectal swabs between participants with rectitis (N=15) and without rectitis (N=62). C and D: Comparison of the peak viral load C) and viral shedding dynamics (D) of MPXV in rectal swabs between participants with HIV (N=30) and without HIV (N=32) among the participants without rectitis. E and F: Comparison of the peak viral load (E) and viral shedding dynamics (F) of MPXV in rectal swab between participants with rectitis (N=12) and without rectitis (N=30) among the participants with HIV. Viral load is expressed as $\log_{10}$ copies per mL. Statistical significance was measured using a Mann-Whitney two-sided test U-test, and p values less than 0.05 were considered statistically significant.

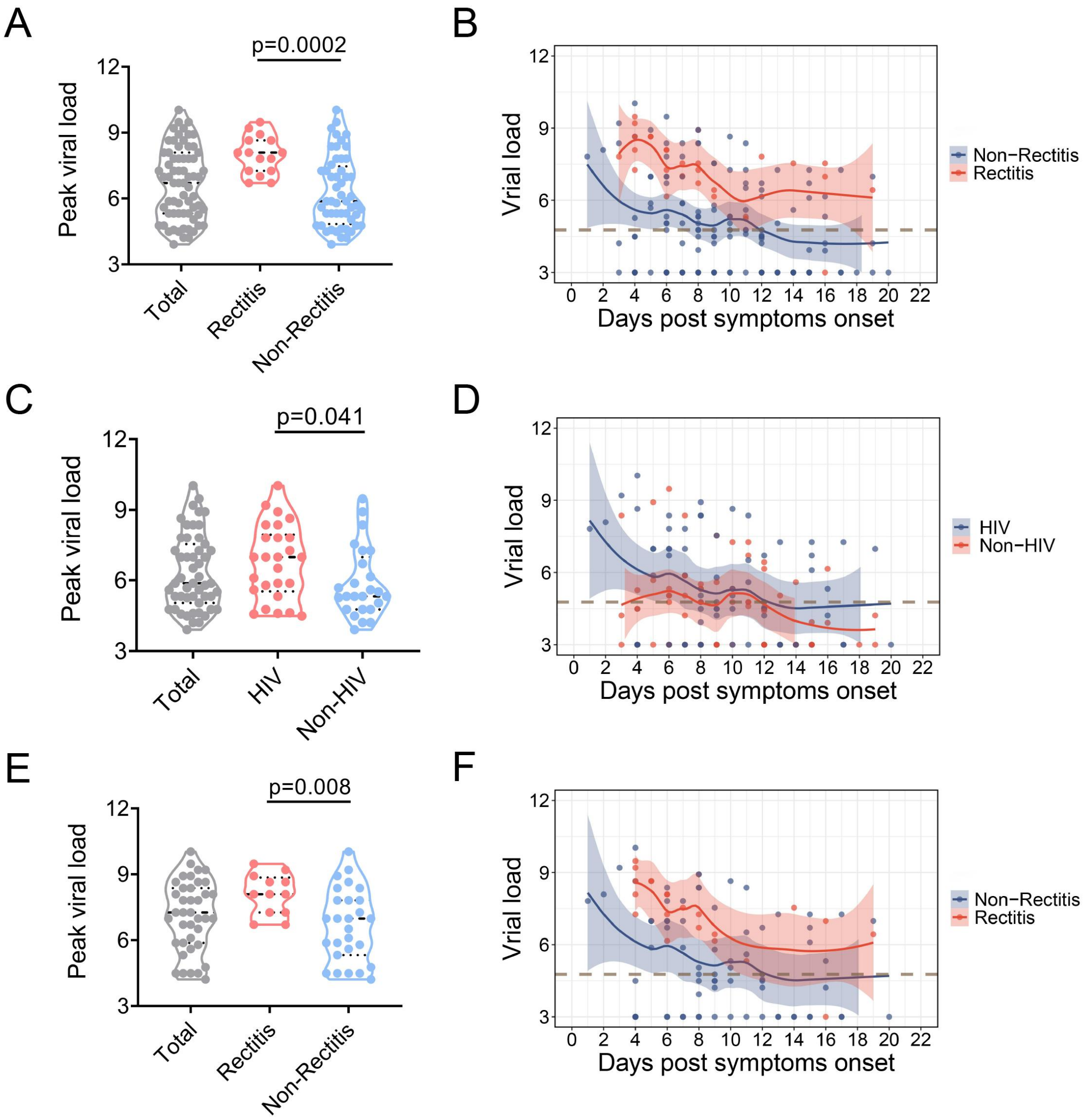

Table S1. Detection of MPXV DNA in multiple sites of Mpox cases during disease progression.

Collection time-points	Sample types	Monkeypox virus infections			
		Total	HIV-positive (N=42)	HIV-negative (N=35)	p values
**Overall**					
**Positive rate (%, n/N)[&]**	Oropharynx	78.95 (60/76)	78.57 (33/42)	79.41 (27/34)	>0.9999
	Saliva	83.78 (62/74)	87.18 (34/39)	80.00 (28/35)	0.5311
	Rectum	88.16 (67/76)	92.86 (39/42)	82.35 (28/34)	0.2841
	Skin lesions*	100.00 (76/76)	100.00 (42/42)	100.00 (34/34)	>0.9999
	Urine	55.26 (42/76)	56.10 (23/41)	54.29 (19/35)	>0.9999
	Plasma	31.17 (24/77)	38.10 (16/42)	22.86 (8/35)	0.1465
**1~7 d.p.o**					
**Positive rate (%, n/N)**	Oropharynx	71.21 (47/66)	65.85 (27/41)	80.00 (20/25)	0.2703
	Saliva	74.58 (44/59)	74.29 (26/35)	75.00 (18/24)	>0.9999
	Rectum	75.41 (46/61)	78.38 (29/37)	70.83 (17/24)	0.5527
	Skin lesions	100.00 (74/74)	100.00 (49/49)	100.00 (25/25)	>0.9999
	Urine	47.37 (27/57)	42.42 (14/33)	54.17 (13/24)	0.4295
	Plasma	24.56 (14/57)	33.33 (11/33)	12.50 (3/24)	0.1183
**Viral loads[#] (median; range)**	Oropharynx	5.32 (4.77-5.88)	5.32 (4.77-6.29)	5.35 (4.77-5.88)	0.2563
	Saliva	6.15 (5.05-6.98)	6.02 (5.10-7.18)	6.35 (5.18-6.65)	0.7185
	Rectum	7.26 (5.88-8.37)	7.82 (6.98-8.65)	5.32 (5.05-7.82)	0.0150
	Skin lesions	7.82 (7.26-8.37)	7.82 (7.26-8.09)	8.09 (7.68-8.65)	0.3717
	Urine	4.77 (4.22-6.24)	4.80 (4.22-6.29)	4.49 (4.22-6.04)	0.7418
	Plasma	4.82 (4.30-5.13)	4.91 (4.30-5.07)	4.74 (4.33-4.96)	0.7012
**8~14 d.p.o**					
**Positive rate (%, n/N)**	Oropharynx	56.00 (42/75)	52.38 (22/42)	60.61 (20/33)	0.4936
	Saliva	69.86(51/73)	76.92 (30/39)	61.76 (21/34)	0.2040
	Rectum	72.73 (56/77)	76.74 (33/43)	67.65 (23/34)	0.4438
	Skin lesions	95.95 (71/74)	95.24 (40/42)	96.88 (31/32)	>0.9999
	Urine	39.19 (29/74)	43.90 (18/41)	33.33 (11/33)	0.4731
	Plasma	14.44 (13/90)	13.21 (7/53)	16.22 (6/37)	0.7649
**Viral loads (median; range)**	Oropharynx	5.32 (4.73-6.15)	5.30 (4.71-6.13)	5.46 (4.69-6.29)	0.8400
	Saliva	5.60 (4.77-6.71)	6.02 (4.49-6.92)	5.60 (5.05-5.88)	0.5962
	Rectum	5.42 (4.77-7.26)	5.60 (4.55-7.26)	5.32 (4.77-6.57)	0.2525
	Skin lesions	7.26 (6.43-8.37)	7.12 (6.26-8.37)	7.26 (6.57-8.29)	0.7125
	Urine	5.32 (5.05-6.04)	5.32 (4.66-5.93)	5.32 (5.05-6.33)	0.1999
	Plasma	4.30 (4.19-4.58)	4.38 (4.30-4.69)	4.22 (4.10-4.41)	0.1353
**15~21 d.p.o**					
**Positive rate (%, n/N)**	Oropharynx	25.00 (6/24)	26.67 (4/15)	22.22 (2/9)	>0.9999
	Saliva	56.52 (13/23)	57.14 (8/14)	55.56 (5/9)	>0.9999
	Rectum	58.33 (14/24)	60.00 (9/15)	55.56 (5/9)	>0.9999

	Skin lesions	91.67 (22/24)	93.33 (14/15)	88.89 (8/9)	>0.9999
	Urine	39.13 (9/23)	20.00 (3/15)	75.00 (6/8)	0.0228
	Plasma	5.71 (2/35)	10.53 (2/19)	0.00 (0/16)	0.4891
**Viral loads (median; range)**	Oropharynx	5.71 (5.16-6.49)	6.13 (5.41-7.07)	5.46 (5.24-5.66)	0.4456
	Saliva	5.05 (4.49-6.98)	5.85 (4.49-7.12)	5.05 (4.22-5.05)	0.8951
	Rectum	6.29 (4.49-6.98)	6.71 (6.10-6.98)	4.22 (3.94-6.15)	0.1088
	Skin lesions	6.71 (5.66-7.73)	6.71 (5.43-7.73)	6.71 (5.74-7.47)	0.3914
	Urine	4.77 (4.49-6.43)	4.49 (4.22-5.77)	4.91 (4.55-6.07)	0.7896
	Plasma	5.57 (5.49-5.63)	5.57 (5.49-5.63)	NA	NA

[&]Individuals with positive detection of MPXV DNA in the indicated sample types.

[#]Viral load measures are expressed as log_{10} copies per mL (mL of viral transport media for lesion, pharynx, and rectum, and mL of urine and plasma)

*Include swabs of lesion surface and exudate.

NA: Not available.

d.p.o: Days post symptoms onset.

Table S2. Collection of the environmental samples.

Sample types	Total	1~7 d.p.o	8~14 d.p.o	15~21 d.p.o
Floor (N)	89	16	55	18
Call button (N)	125	31	70	24
Light switch (N)	124	30	70	24
Television remote control (N)	94	21	55	18
Bed handrail (N)	124	31	70	23
Bedside cupboard (N)	125	31	70	24
Chair (arm rest) (N)	107	30	58	19
Door handle (patient room to bathroom) (N)	93	22	54	17
Deposition area (Air conditioning air outlet) (N)	93	20	55	18
Mobile phone (N)	125	31	70	24
Clothes (N)	125	31	70	24
Pillow (N)	125	31	70	24
Toilet flush handle (N)	94	21	55	18
Shower handle (N)	95	21	55	19
Delivery window (N)	95	21	55	19

d.p.o: Days post symptoms onset.

Table S3. Detection of MPXV DNA on different environmental fomits during disease progression.

Characteristics	Overall	Days post symptoms onset (d.p.o)			p values$		
		1~7	8~14	15~21	1~7 vs 8~14	1~7 vs 15~21	8~14 vs 15~21
**Median viral loads of surfaces in patients' room# (IQR)**							
Floor	5.54 (5.06-6.26)	5.21 (5.08-5.92)	5.56 (4.96-6.27)	5.86 (5.26-6.65)	0.7353	0.5013	0.8562
Call button	5.39 (4.87-6.16)	5.45 (4.89-5.85)	5.30 (4.81-6.07)	5.75 (5.17-6.39)	0.9996	0.6143	0.5015
Light switch	4.85 (4.57-5.43)	5.20 (4.60-5.40)	4.84 (4.56-5.29)	5.14 (4.57-5.50)	0.1630	0.8094	0.5173
Television remote control	5.26 (4.85-5.84)	4.90 (4.53-5.20)	5.28 (4.83-5.90)	5.38 (5.07-5.95)	0.4800	0.2762	0.7542
Bed handrail	4.93 (4.69-5.64)	4.94 (4.73-5.71)	5.00 (4.71-5.59)	4.89 (4.56-5.61)	0.8688	0.9822	0.9747
Bedside cupboard	5.47 (4.73-6.35)	5.57 (4.71-6.14)	5.72 (4.84-6.49)	5.02 (4.63-6.00)	09435	0.8848	0.5423
Chair (arm rest)	5.02 (4.77-5.64)	4.89 (4.46-5.10)	5.31 (4.64-5.67)	5.30 (4.93-5.90)	0.5176	0.1077	0.3665
Door handle (patient room to bathroom)	5.34 (4.90-6.03)	5.03 (4.94-5.22)	5.27 (4.84-6.17)	6.01 (5.13-6.04)	0.4802	0.2358	0.7268
Deposition area (Air conditioning air outlet)	5.53 (5.05-6.52)	5.45 (5.17-6.25)	5.47 (5.01-6.50)	5.95 (5.17-6.91)	0.9982	0.8002	0.6700
**Median viral loads of patients' daily living equipment (IQR)**							
Mobile phone	5.22 (4.84-5.83)	5.04 (4.84-5.33)	5.23 (4.78-5.89)	5.31 (4.92-5.84)	0.5769	0.5199	0.9377
Clothes	5.12 (4.68-5.79)	5.06 (4.58-5.29)	5.16 (4.76-5.86)	5.15 (4.89-5.79)	0.5649	0.5515	0.9676
Pillow	5.32 (4.85-6.07)	5.08 (4.50-6.00)	5.36 (4.94-6.15)	4.98 (4.66-5.85)	0.9036	0.9522	0.7151
**Median viral loads of surfaces in bathroom (IQR)**							

Toilet flush handle	5.12 (4.74-6.01)	5.54 (4.85-5.88)	5.13 (4.71-6.05)	4.92 (4.90-5.80)	0.8960	0.7586	0.9054
Shower handle	5.24 (4.87-5.64)	5.45 (5.23-5.61)	5.18 (4.64-5.49)	5.30 (5.00-5.67)	0.1439	0.4991	0.8645
**Median viral loads of surfaces in delivery window (IQR)**	5.17 (4.74-5.63)	4.69 (4.53-5.04)	5.17 (4.74-5.69)	5.34 (4.92-5.83)	0.6679	0.4201	0.7239

[#]Viral load measures are expressed as $\log_{10}$ copies per mL (mL of viral transport media)

[$]Statistical significance was measured using a Mann-Whitney two-sided test U-test.

IQR: Interquartile range.

NA: Not available.
