# Supplementary material for: Longitudinal viral shedding and antibody response characteristics of men with acute infection of monkeypox virus: a prospective cohort study
Source: Nat Commun. 2024 May 27;15:4488. doi: 10.1038/s41467-024-48754-8 (PMC11130326; doi:10.1038/s41467-024-48754-8)
Supplement: Supplementary file 3 — Reporting Summary [file 41467_2024_48754_MOESM3_ESM.pdf]

nature portfolio

Corresponding author(s): Yingxia Liu

Last updated by author(s): May 1, 2024

Reporting Summary

Nature Portfolio wishes to improve the reproducibility of the work that we publish. This form provides structure for consistency and transparency in reporting. For further information on Nature Portfolio policies, see our Editorial Policies and the Editorial Policy Checklist.

Statistics

For all statistical analyses, confirm that the following items are present in the figure legend, table legend, main text, or Methods section.

n/a	Confirmed	
☐	☒	The exact sample size (*n*) for each experimental group/condition, given as a discrete number and unit of measurement
☐	☒	A statement on whether measurements were taken from distinct samples or whether the same sample was measured repeatedly
☐	☒	The statistical test(s) used AND whether they are one- or two-sided<br>*Only common tests should be described solely by name; describe more complex techniques in the Methods section.*
☒	☐	A description of all covariates tested
☒	☐	A description of any assumptions or corrections, such as tests of normality and adjustment for multiple comparisons
☐	☒	A full description of the statistical parameters including central tendency (e.g. means) or other basic estimates (e.g. regression coefficient) AND variation (e.g. standard deviation) or associated estimates of uncertainty (e.g. confidence intervals)
☐	☒	For null hypothesis testing, the test statistic (e.g. *F*, *t*, *r*) with confidence intervals, effect sizes, degrees of freedom and *P* value noted<br>*Give P values as exact values whenever suitable.*
☒	☐	For Bayesian analysis, information on the choice of priors and Markov chain Monte Carlo settings
☒	☐	For hierarchical and complex designs, identification of the appropriate level for tests and full reporting of outcomes
☒	☐	Estimates of effect sizes (e.g. Cohen's *d*, Pearson's *r*), indicating how they were calculated

Our web collection on statistics for biologists contains articles on many of the points above.

Software and code

Policy information about availability of computer code

Data collection	No software was used for data collection.
Data analysis	We used SPSS 20.0 for Windows, GraphPad Prism 8.0 and R software 4.04 for the analysis of the data.

For manuscripts utilizing custom algorithms or software that are central to the research but not yet described in published literature, software must be made available to editors and reviewers. We strongly encourage code deposition in a community repository (e.g. GitHub). See the Nature Portfolio guidelines for submitting code & software for further information.

Data

Policy information about availability of data

All manuscripts must include a data availability statement. This statement should provide the following information, where applicable:

- Accession codes, unique identifiers, or web links for publicly available datasets
- A description of any restrictions on data availability
- For clinical datasets or third party data, please ensure that the statement adheres to our policy

The data supporting the findings of this study are available within the paper and the supplementary information files. Source data are provided with this paper.

Research involving human participants, their data, or biological material

Policy information about studies with human participants or human data. See also policy information about sex, gender (identity/presentation), and sexual orientation and race, ethnicity and racism.

Reporting on sex and gender	The 'Male' reported in Table 1 is based on the standard of 'sex', i.e., defined based on currently understood biological differences between females and males, including chromosomes, sex organs, and endogenous hormonal profiles.
Reporting on race, ethnicity, or other socially relevant groupings	All the participants were Chinese Han population.
Population characteristics	Totally, 77 hospitalized patients with laboratory confirmation of MPXV infection were enrolled in this study with a median age of 30 years, and only 5 patients received smallpox vaccination during childhood. Of note, 72 patients were men who have sex with men (MSM), and the rest bisexual.
Recruitment	A total of 77 men with acute infection of MPXV admitted to Shenzhen Third People's Hospital during June 9, 2023 to November, 5, 2023 (N=77) (Shenzhen, Guangdong, China) for both isolation and treatment were enrolled in this study. All the patients were included if they are willing to participant in this study, without any selection.
Ethics oversight	The study protocol was approved by the Ethics Committees of Shenzhen Third People's Hospital, and written informed consents were obtained.

Note that full information on the approval of the study protocol must also be provided in the manuscript.

Field-specific reporting

Please select the one below that is the best fit for your research. If you are not sure, read the appropriate sections before making your selection.

☒ Life sciences ☐ Behavioural & social sciences ☐ Ecological, evolutionary & environmental sciences

For a reference copy of the document with all sections, see nature.com/documents/nr-reporting-summary-flat.pdf

Life sciences study design

All studies must disclose on these points even when the disclosure is negative.

Sample size	A total of 77 men with acute infection of MPXV admitted to Shenzhen Third People's Hospital during June 9, 2023 to November, 5, 2023 (N=77) (Shenzhen, Guangdong, China) for both isolation and treatment were enrolled in this study. The sample size was based on availability of patients admitted to our hospital. No prior sample size calculation was performed. According to some published studies (PMID: 36521505, PMID: 336183707, PMID: 35623380, etc.), this sample size is sufficient for evaluating the viral and antibody dynamics.
Data exclusions	No data were excluded from the analyses.
Replication	The MPXV surface proteins specific IgG antibodies in plasma specimens were detected using Enzyme-linked immunosorbent assays (ELISA) as previously reported and run in triplicate. All attempts at replication were successful.
Randomization	Randomization was not relevant to this study as this was an observational study.
Blinding	Blinding was not relevant to this study because of the observational design.

Reporting for specific materials, systems and methods

We require information from authors about some types of materials, experimental systems and methods used in many studies. Here, indicate whether each material, system or method listed is relevant to your study. If you are not sure if a list item applies to your research, read the appropriate section before selecting a response.

Materials & experimental systems

n/a	Involved in the study
☐	☒ Antibodies
☐	☒ Eukaryotic cell lines
☒	☐ Palaeontology and archaeology
☒	☐ Animals and other organisms
☒	☐ Clinical data
☒	☐ Dual use research of concern
☒	☐ Plants

Methods

n/a	Involved in the study
☒	☐ ChIP-seq
☒	☐ Flow cytometry
☒	☐ MRI-based neuroimaging

Antibodies

Antibodies used

Horseradish peroxidase (HRP)-conjugated goat anti-human IgG antibody (Sangon Biotech, Cat: D110150-0100, 1:10000 dilution); HRP-conjugated Vaccinia Virus Polyclonal Antibody (Invitrogen, Cat: PA1-73192, 1:500 dilution);

Validation

All the antibodies used in this study were commercial antibodies and were only used for applications, with validation procedures described on the following sites of the manufacturers:
Horseradish peroxidase (HRP)-conjugated goat anti-human IgG antibody: https://store.sangon.com/productDetail?productInfo.code=D110150
HRP-conjugated Vaccinia Virus Polyclonal Antibody: https://www.thermofisher.cn/cn/zh/antibody/product/Vaccinia-Virus-Antibody-Polyclonal/PA1-73192

Eukaryotic cell lines

Policy information about cell lines and Sex and Gender in Research

Cell line source(s)

Vero E6 cells were obtained from ATCC.

Authentication

The cell line was frequently checked for cellular morphologies, growth rates and functions.

Mycoplasma contamination

All cell lines used were tested (by PCR) and were mycoplasma free.

Commonly misidentified lines
(See ICLAC register)

No commonly misidentified cell lines were used.

Plants

Seed stocks

Not applicable.

Novel plant genotypes

Not applicable.

Authentication

Not applicable.
